# Editorial: Integrating omics to unveil legume stress response mechanisms

**DOI:** 10.3389/fpls.2025.1583412

**Published:** 2025-05-07

**Authors:** Inês Maria Valente, Cristiano Soares, Henrique Trindade

**Affiliations:** ^1^ REQUIMTE, LAQV, ICBAS, School of Medicine and Biomedical Sciences, University of Porto, Porto, Portugal; ^2^ REQUIMTE, LAQV, Department of Chemistry and Biochemistry, Faculty of Sciences, University of Porto, Porto, Portugal; ^3^ GreenUPorto – Sustainable Agrifood Production Research Centre/Inov4Agro, Biology Department, Faculty of Sciences of University of Porto, Porto, Portugal; ^4^ Centre for the Research and Technology of Agro-Environmental and Biological Sciences (CITAB), University of Trás-os-Montes and Alto Douro, Real, Portugal

**Keywords:** abiotic stress, biotic stress, agriculture, omics, legumes

Legumes (Fabaceae) are among the most important crop families globally, with significant economic relevance for both human and animal consumption. These plants are crucial for balancing animal and plant-based protein intake, contributing to healthier and more sustainable diets. The resilience of legume crops to various edaphoclimatic conditions, coupled with their ability to biologically fix atmospheric nitrogen, makes them essential for sustainable agricultural systems. Indeed, the high diversity within the Fabaceae family allows different species and cultivars to adapt to a wide range of soil and climate environments, enabling their cultivation across diverse agroclimatic regions.

The expected increase of the World population and the intensification of climate change-related impacts in the next decades will translate into unprecedent pressures on the agricultural sustainability and profitability. Besides the economic impact on industries dependent on production, the food/feed security and availability will also be severely affected by future global scenarios, especially in already susceptible regions, such as the Mediterranean. Indeed, while it is necessary that there is an increase in crop produtivity, this growth must be accompanied by increasingly sustainable practices and the use of fewer natural resources. Therefore, the United Nations 2030 Agenda for Sustainable Development clearly highlights, as a top priority, the promotion of sustainable production systems and resilient practices that foster increased productivity and combat climate change.

Over the past decades, the effects of drought, the most limiting factor for plant growth, have been extensively studied, along with the development of stress mitigation strategies. Being an important source of plant protein, studies exploring the responses of lentil (*Lens culinaris* Medik.) to water-deficit conditions are particularly important. In this Research Topic (RT), a review on lentil’s adaptation to drought and the current approaches used to enhance its stress tolerance was published by Noor et al. In their work, authors discussed the mechanisms, morpho-physiological, biochemical and molecular adaptations of lentil plants to counteract water-deficit conditions. Moreover, Noor et al. reviewed the recent progress in genotype screening and breeding for drought-stress tolerance in this species, by means of omics-based approaches (genomics, proteomics, phenomics, ionomics, transcriptomics, metabolomics, and miRNA-omics), ultimately aiming the development of drought-tolerant varieties.

When referring to crop improvement and maximizing agricultural yields, other approaches, besides traditional breeding and genome editing, have been widely studied in the last decades. Particularly with regard to drought-ameliorating strategies, the application of fertilizers and biostimulants for the purpose of mitigating the negative effects of water deficit has been thoroughly studied. In this RT, Fozi et al., unravelled the impact of a seaweed extract on the phytochemical characteristics and antioxidant activity of licorice (*Glycyrrhiza glabra* L.). Seaweed extracts are recognized to stimulate the synthesis of antioxidant enzymes and phenolic compounds, upregulate genes involved in abscisic acid (ABA) signaling and antioxidant pathways. Fozi et al. also observed that the presence of seaweed extract in water stress conditions enhanced glycyrrhizic acid and glabridin contents, and increased the activity of ascorbate peroxidase, catalase, peroxidase, and superoxide dismutase, contributing to a more efficient activation of the plants’ antioxidant mechanisms. The findings of this study suggest that seaweed can enhance the water stress tolerance of licorice by increasing antioxidant enzymes and metabolites contents, paving the way to the use of algal species to improve the resilience of licorice crops, among others. In fact, the beneficial role of macro and microalgae-related compounds (e.g., aqueous and organic extracts, composts, dry biomass) has been increasingly recognized, although the exact mechanisms underlying such positive effects still remain elusive.

Within this context, he integration of omics technologies is a promising approach to identify genetic, transcriptomic and phenotypic pathways involved in biotic and abiotic stress tolerance. As sessile organisms, plants are almost permanently exposed to a wide range of abiotic and biotic stresses, such as drought, salinity, UV radiation, extreme temperatures, herbivores, pathogens, and adverse soil conditions, which can largely hamper their growth and development, by disrupting key metabolic and physiological events. Metabolomics is a powerful tool for interpreting biotic and abiotic stress tolerance in plants, by providing a detailed glimpse of plant species’ metabolite composition and how these organisms may change their metabolome according to the environment. Over the past decade, significant advancements in instrumentation and bioinformatics tools have enhanced the capabilities and workflow of metabolomics, enabling comprehensive scanning of plant metabolomes. Different metabolites that have never been investigated can now be identified and quantified through metabolomics and other associated omics, thus providing a promising link to bridge the gap between genotype and morphological/physiological traits. Despite the characterization of approximately 200,000 compounds in the Plantae kingdom, there remains a need for a deeper understanding of the metabolic pathways associated with stress responses in legumes. Recent advancements in high-resolution analytical technologies, such as mass spectrometry, have revitalized metabolomic studies, yet gaps in knowledge still persist. Moreover, the combination of several omic approaches to fully establish the link between gene expression, protein synthesis and metabolism will much likely take the spotlight in the coming years within the field of plant stress physiology. By working on this Research Topic, Guan et al. employed both untargeted metabolomics and transcriptomics to study the regulatory mechanisms involved in the transition from endo- to ecodormancy in the buds of the Chinese herbal medicine *Astragalus membranaceus* (Fisch.) Bge. var. mongholicus (Bge.) Hsiao. This plant is widely used in Asia and is cultivated by transplanting the annual rhizomes. Understanding the dormancy mechanisms of underground buds can help farmers to determine the optimal periods for harvesting and transplanting rhizomes, ensuring better survival and growth rates. In this work, the researchers identified 1069 differentially accumulated metabolites (DAMs) and 16832 differentially expressed genes (DEGs) during the dormancy transition, primarily involved in amino acid and carbohydrate metabolism, hormone signaling pathways, and starch and sucrose metabolism. They have also demonstrated that indole-3-acetic acid (IAA) may play a key role in the transition from endo- to ecodormancy, as the highest IAA content and the highest number of DEGs enriched in the IAA signaling pathway. These insights into the molecular mechanisms underlying bud dormancy in *Astragalus membranaceus* open a new approach over strategies for breaking dormancy in advance and shortening breeding time, while also clearly demonstrating the importance of combining complementary approaches to fully elucidate complex biological phenomena.

Also exploring the potential of omic-based strategies, the tungsten-stress response of soybean (*Glycine max*) in symbiotic relationships with Rhizobium bacteria was characterized by Preiner et al. through comprehensive metabolomic and proteomic analyses. Main findings revealed that symbiosis increased the synthesis of protective compounds with antioxidant properties in plants, which ultimately translated into distinct metabolomic and proteomic landscapes from nitrogen-fertilized soybeans. The symbiotically grown plants developed a specific metabolic adjustment for an improved tolerance to stress, a valuable strategy to enhance crop productivity and resilience particularly in environments contaminated by heavy metals.

Lastly, on a different perspective, a metabolomics-based approach was implemented to unravel how microplastic exposure could alter the allelopathic effects between native and invasive plant species (Yuan et al.). Currently, environmental contamination by microplastics and their effects on ecological health and sustainability has emerged as a hot Research Topic, though their effects on plant physiology and metabolism remain mostly unknown. Yuan et al. have found that polyethylene microplastics can influence the co-existence of invasive and native plants by altering their allelopathic potential, inducing a negative allelopathic effect for the native plant *Achyranthes* on co-occurring plants, mediated through changes in leaf metabolites. This research provided a novel perspective on how microplastic pollution can modify plant species interactions and ecosystem dynamics, crucial for managing invasive species and protecting native biodiversity.

Overall, this RT gathers novel findings on how legume plants might counteract and adapt to different environmental constraints, while also showing the potential of omics-based approaches in such responses. The research presented ultimately offers significant contributions to developing new biotechnological applications and breeding programs towards enhanced crop resilience and improved agricultural practices and sustainability. This RT highlights the revolution on crop management and improvement provided by omics approaches in the context of the current challenges of agricultural systems.

